# IL-12p40 Monomer: A Potential Player in Macrophage Regulation

**DOI:** 10.3390/immuno4010005

**Published:** 2024-02-23

**Authors:** Brian Jeong, Kalipada Pahan

**Affiliations:** 1 Department of Neurological Sciences, Rush University Medical Center, Chicago, IL 60612, USA; 2 Division of Research and Development, Jesse Brown Veterans Affairs Medical Center, Chicago, IL 60612, USA

**Keywords:** macrophage, IL-12p40 monomer, IL-10, TNFα, immunomodulation

## Abstract

Macrophages are myeloid phagocytic leukocytes whose functions are to protect against infections, mediate T-cell responses, and maintain tissue homeostasis. IL-12p40 monomer is a cytokine that is largely produced by macrophages, and it has, for the longest time, been considered a largely non-functional cytokine of the IL-12 family. However, new research has emerged that demonstrates that this p40 monomer may play a bigger role in shaping immune environments. To shed light on the specific effects of p40 monomer on macrophages and their surrounding environment, we showed, through cell culture studies, qPCR, ELISA, and immunofluorescence analyses, that the direct administration of recombinant p40 monomer to RAW 264.7 cells and primary lung macrophages stimulated the production of both pro-inflammatory (TNFα) and anti-inflammatory (IL-10) signals. Accordingly, p40 monomer prevented the full pro-inflammatory effects of LPS, and the neutralization of p40 monomer by mAb a3-3a stimulated the pro-inflammatory effects of LPS. Furthermore, we demonstrated that the intranasal administration of p40 monomer upregulated TNFα^+^IL-10^+^ macrophages in vivo in the lungs of mice. Collectively, these results indicate an important immunoregulatory function of p40 monomer in the upregulation of both pro- and anti-inflammatory molecules in macrophages.

## 1. Introduction

Macrophages are phagocytic leukocytes of myeloid lineage of the innate immune system that are responsible for protecting against infectious pathogens, producing inflammatory and/or anti-inflammatory effector proteins, and clearing cellular debris [1-3]. Like other leukocytes, macrophages have demonstrated plasticity and the ability to adapt to external environmental stimuli through interaction with signaling proteins, adjacent cells, and underlying extracellular matrix proteins [1-4]. Activated macrophages are often categorized as either M1 or M2 cells [5-7]. M2 macrophages tend to produce anti-inflammatory signals and are commonly associated with wound repair and Th2-mediated effects via signals such as IL-10, VEGF, TGF-β, and ornithine [8,9]. On the other hand, M1 denotes classically activated macrophages, exhibiting pro-inflammatory characteristics associated with the pathogenic invasion and activation of Th1-mediated immune response [5-7,10]. M1 macrophages carry this out through the production of signals such as IL-12p70, IL-6, TNFα, and nitric oxide (NO), with IL-12p70 being considered one of the most potent pro-inflammatory cytokines [11-13].

Functional IL-12 exists as a heterodimer composed of p40 and p35 [11,14]. However, p40 is known not only as a subunit of IL-12 but also as a subunit of IL-23 (along with subunit p19), as a homodimer, and as a monomer [15,16]. Initially, only IL-12 and IL-23 were considered bioactive, and p40 homodimer was thought of as an antagonist for IL-12 [17]. Since all four cytokines (IL-12, IL-23, p40 homodimer, and p40 monomer) contain p40 in different forms, without using a specific monoclonal antibody (mAb), it was not possible to characterize the functions of p40 homodimer and p40 monomer. However, mAbs against p40 monomer and p40 homodimer were not available. Therefore, we have generated specific functional blocking mAbs against p40 monomer and p40 homodimer separately [18] and found that functional blocking monoclonal antibodies against p40 homodimer protect mice from experimental autoimmune encephalomyelitis (EAE), an animal model of multiple sclerosis (MS) [19], indicating an important role of p40 homodimer in the disease process of EAE and MS. While the functions of p40 monomer are not known, by using a specific mAb, recently, we have delineated the selective upregulation of p40 monomer, but not that of IL-12, IL-23, and p40 homodimer, in the serum of prostate cancer and breast cancer patients compared with healthy controls [20,21]. Moreover, we have also seen a decrease in p40 monomer and an increase in IL-12, IL-23, and p40 homodimer in the serum of MS patients compared with healthy controls [11]. However, the functions of p40 monomer remain poorly understood. Therefore, we aimed to shed some light on the possible direct effects of p40 monomer on macrophages. Here, we describe that while p40 monomer was capable of upregulating both pro-inflammatory TNFα and anti-inflammatory IL-10 in macrophages, the pro-inflammatory effects of LPS were modestly inhibited by recombinant p40 monomer and stimulated by the mAb-mediated neutralization of p40 monomer.

## 2. Materials and Methods

### 2.1. Cell Culture and Reagents

The RAW 264.7 macrophage cell line was purchased from ATCC, Manassas, VA, USA. Recombinant p40 monomer and antibodies against iNOS were obtained from BD Biosciences (Franklin Lakes, NJ, USA). DNase I, collagenase D, and LPS were obtained from Millipore-Sigma (Burlington, MA, USA). An Armenian hamster isotype was obtained from Tonbo Bioscience (San Diego, CA, USA). Primary antibodies for TNFα were obtained from Santa Cruz Biotechnology (Dallas, TX, USA). Antibodies for IL-10 and CD68 were obtained from ThermoFisher (Waltham, MA, USA). Secondary fluorophore-conjugated antibodies were obtained from Jackson Immunoresearch (West Grove, PA, USA).

### 2.2. Culturing of RAW 264.7 Macrophage Cells

RAW 264.7 cells were cultured in DMEM (ThermoFisher, Waltham, MA, USA) containing 10% FBS (Atlas Biologicals, Fort Collins, CO, USA) and 1% penicillin/streptomycin (ThermoFisher, Waltham, MA, USA). Experiments were conducted in 12-, 24-, and 6-well plate formats with a plating density of 100,000 cells/mL. Treatments were carried out in serum-free medium to eliminate any effect from serum proteins.

### 2.3. Isolation of Primary Lung Macrophages

Primary alveolar and interstitial macrophages were collected from the lungs of 2–4-month-old C57BL/6 mice. All steps were performed in a cell culture hood under sterile conditions to prevent contamination. Lungs were perfused with cold PBS by injecting it into the right ventricle of the heart. Single-cell suspensions were obtained by homogenizing and incubating tissue homogenates with DNase I and collagenase D for 1 h at 37 °C. The cells were then strained through a 40 μm cell strainer (Cell Treat; Peperell, MA, USA), spun down at 300 g for 5 min, resuspended in an RBS lysis buffer (eBioscience, San Diego, CA, USA), and spun down again, and the resulting single-cell suspension was plated. Cells were allowed to adhere and grow for 5–6 days. Trypsin at 0.05% (ThermoFisher, Waltham, MA, USA) was used to detach loosely adherent lung macrophages, which were plated for treatment. These cells were homogeneously stained for CD68.

### 2.4. ELISA

Supernatants from treated RAW 264.7 cells were used to measure the levels of TNFα, IL-10, and IL-6. Uncoated ELISA kits were purchased from ThermoFisher (Waltham, MA, USA), and assays were performed according to the manufacturer’s protocols. Coated Quantikine ELISA kits from R&D (Mckinley Place, NE, USA) were utilized for mouse IFNβ measurements according to the manufacturer’s protocol.

### 2.5. Immunofluorescence

Immunofluorescence was performed as described previously [11]. Briefly, RAW 264.7 cells and primary lung macrophages were plated onto cover slips. After treatment, cells were fixed with ice-cold 4% paraformaldehyde diluted with PBS. Permeabilization was carried out with 0.5% Triton-X diluted in PBS. The wells were then blocked with 3% BSA in PBS for 1 h at room temperature followed by incubation with primary antibodies at 4 °C overnight. Cells were then washed and incubated with the appropriate fluorophore-conjugated secondary antibodies at room temperature for 1 h. Cells were washed again and incubated with DAPI at 1:10,000 for 5 min. Coverslips were removed, mounted with cytoseal, and observed with a BX-42 Olympus microscope.

### 2.6. Intranasal Administration of Recombinant p40 Monomer

C57BL/6 female mice (4–6-months old) were treated intranasally with recombinant p40 monomer (50 ng/mouse/day). The p40 monomer dissolved in 2 μL of sterile PBS was delivered through one nostril via a Pipetman pipette while keeping the mouse in supine position. After 4 days of treatment, lung tissues were collected for further studies.

### 2.7. RNA Extraction and Quantitative Real-Time PCR (qPCR)

RNA from tissue and cells was collected using the RNeasy Plus Mini kit obtained from Qiagen (Germantown, MA, USA). RNA extraction was performed according to the manufacturer’s protocol as previously described [11,21]. After extraction, cDNA was synthesized using reagents obtained from ThermoFisher (Waltham, MA, USA). The qPCR was performed using SYBR Green and measured using the Quantstudio 3 system from ThermoFisher (Waltham, MA, USA). Ct values were collected, and fold-change values, calculated using the delta–delta method of analysis.

### 2.8. FACS Analysis

Primary cells collected from the lungs were stained for flow cytometric analyses. Cells were first stained with Aqua live/dead (ThermoFisher, Waltham, MA, USA) to exclude dead cells; then, they were washed with PBS and incubated with Fc block (BD Biosciences, Franklin Lakes, NJ, USA) to control for non-specific binding. Cells were washed and incubated with FITC-conjugated CD68 antibody (Biolegend, San Diego, CA, USA) for 30 min at 4 °C. The samples were then washed and fixed with 4% paraformaldehyde in PBS for 10 min followed by permeabilization with a permeabilization buffer (ThermoFisher, Waltham, MA, USA) for 10 min. The samples were then incubated with APC-conjugated IL-10 antibody and PE-conjugated TNFα antibody (Biolegend, San Diego, CA, USA) in permeabilization buffer for 30 min at 4 °C. Samples were washed twice, resuspended in flow staining buffer (ThermoFisher, Waltham, MA, USA) and examined in the LSRFortessa cell analyzer (BD Biosciences). Results were analyzed using FlowJo V10 software as previously described [11].

### 2.9. Immunoblotting Analysis

Tissue samples were homogenized with RIPA buffer and spun at 10,000 rpm for 10 min at 4 °C. Supernatants (protein samples) were quantified using the DC Protein assay from Bio-Rad (Des Plaines, IL, USA) according to the manufacturer’s protocol, and 50 micrograms of protein per well was run on 12% SDS-PAGE gels. The samples were then transferred onto 0.4 μm nitrocellulose membranes. The membranes were blocked with Licor (Lincoln, NE, USA) blocking buffer and probed overnight with primary antibodies at 4 °C. The following day, the membranes were washed and probed with the appropriate Licor fluorophore-tagged secondary antibodies for 1 h at room temperature. The membranes were then washed and imaged on an Odyssey infrared scanner (Licor, Lincoln, NE, USA) followed by Western blot band analysis using Image Studio Lite V5.2. Results were normalized to the band intensity of β-actin.

### 2.10. Statistical Analysis

Statistical analyses were performed using Graphpad Prism 10.1.1. Statistical differences between means were calculated by the *t*-test (two-tailed). Variance between multiple means was calculated via one-way ANOVA, followed by Tukey’s post hoc tests. The criterion for statistical significance was *p* ≤ 0.05. Values are expressed as means ± SEs. Real-time qPCR heatmaps and clustering hierarchies were generated using the R package pheatmap, version 1.0.12.

## 3. Results

### 3.1. Recombinant p40 Monomer Treatment Induced the Expression of Anti-Inflammatory Cytokine IL-10, Pro-Inflammatory Cytokines, and Different Immunomodulatory Molecules in RAW 264.7 Macrophages

**Figure 1.**
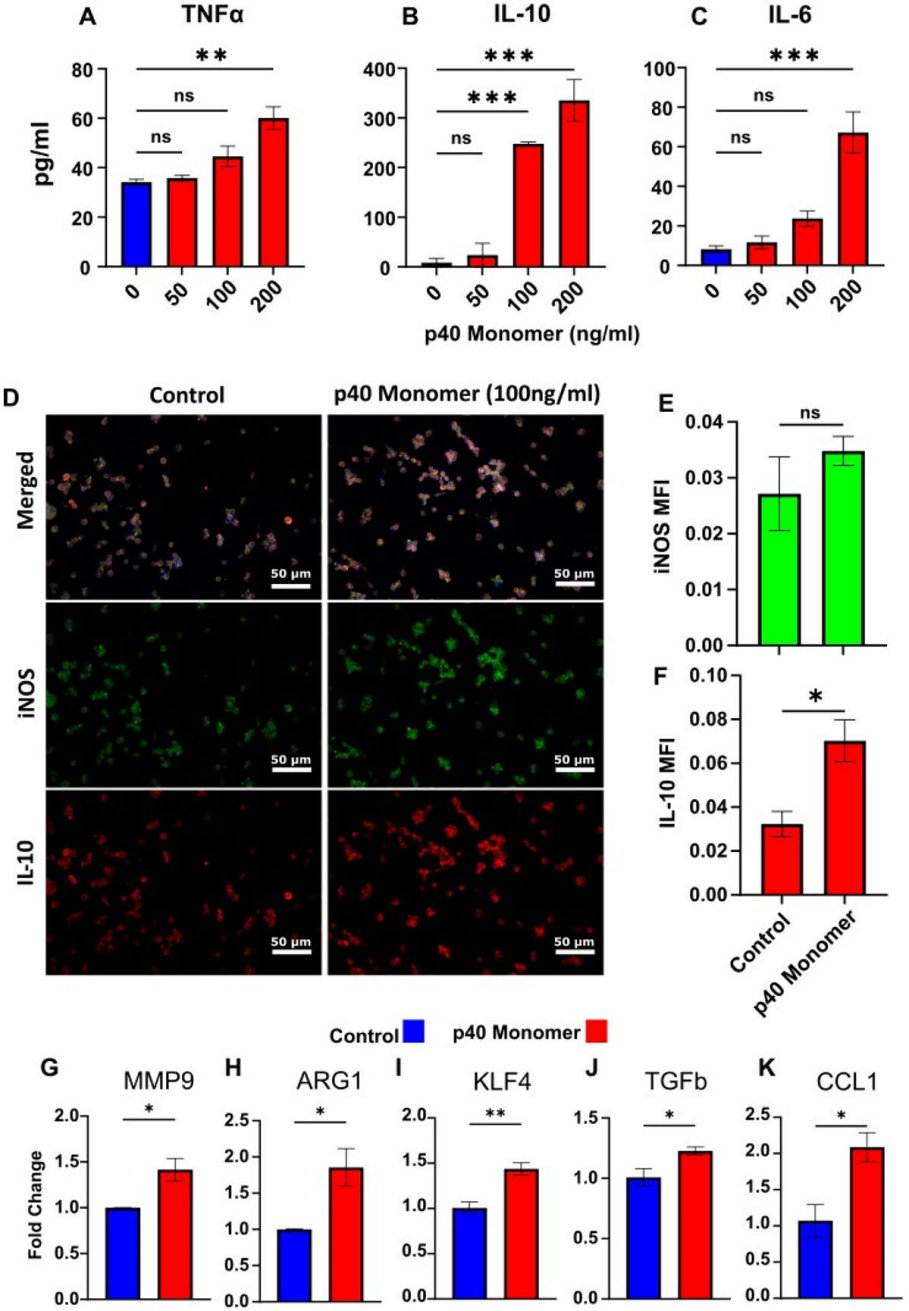
Effects of direct administration of recombinant p40 monomer to RAW 264.7 cells. Cells were treated with various concentrations of p40 monomer once at 70% confluence for 24 h in serum-free medium. ELISA-based cell supernatant cytokine measurement results show levels of increase in TNFα, IL-10, and IL-6 (**A**–**C**). Immunofluorescent imaging demonstrates increases in levels of intracellular iNOS and IL-10 after treatment with 100 ng/mL p40 monomer for 24 h. Images taken at 20× magnification (**D**) and quantitative MFI analysis normalized to DAPI content (**E**,**F**). Real-time PCR results for characteristic markers associated with lung remodeling (MMP9), M2 macrophages (ARG1), cancer-associated phenotype (KLF4, TGFβ), and M2b phenotype (CCL1) (**G**–**K**). Results are means ± SEMs of three different experiments. Significance was determined using one-way ANOVA or two-tailed *t*-tests. * *p* < 0.05; ** *p* < 0.01; *** *p* < 0.001; ns, not significant.

To test the direct effect of p40 monomer on macrophages, cells were treated with different concentrations (50, 100, and 200 ng/mL) of p40 monomer under serum-free conditions for 24 h. When supernatants were analyzed for pro-inflammatory cytokines, we observed a dose-dependent increase in the secretion of both TNFα (Figure 1A) and IL-6 (Figure 1C). However, it was found that the anti-inflammatory signal IL-10 (Figure 1B) also increased commensurately with the concentration of p40 monomer. IL-12p70 was excluded from measurement due to non-specific binding of ELISA antibodies to p40 monomer.

Since significant induction of IL-10 production was found at a dose of 100 ng/ml p40 monomer (Figure 1B), we used this concentration for subsequent experiments. Internal staining was carried out for iNOS (inducible nitric oxide synthase), a common marker for classical M1 macrophages [6], and its expression levels were compared to those of IL-10 (Figure 1D). After treatment with 100 ng/mL p40 monomer, there was a slight increase in iNOS, but it was not significant (Figure 1D,E). However, the detected levels of IL-10 were shown to be significantly upregulated after treatment, again confirming the induction of both pro- and anti-inflammatory signals by p40 monomer (Figure 1D,F).

To further investigate the phenotypic changes that occur after direct treatment with p40 monomer, the mRNA expression of certain immunomodulatory molecules was analyzed via qPCR. Arginase 1 (ARG1) is a marker of classical M2 macrophages, and the qPCR results demonstrated an increase in ARG1 transcription (Figure 1H). On the other hand, increased expression of MMP9 (Figure 1G), KLF4 (Figure 1I), and TGFβ (Figure 1J) induced by p40 monomer could imply a proclivity towards lung remodeling and a tumor-associated macrophage (TAM) phenotype. Furthermore, an increase in CCL1 mRNA (Figure 1K) levels induced by p40 monomer may be indicative of an M2b-specific profile [12].

### 3.2. Treatment of Primary Murine Lung Macrophages with Recombinant p40 Monomer Induced Similar Pro- and Anti-Inflammatory Phenotypes in RAW 264.7 Cells

**Figure 2.**
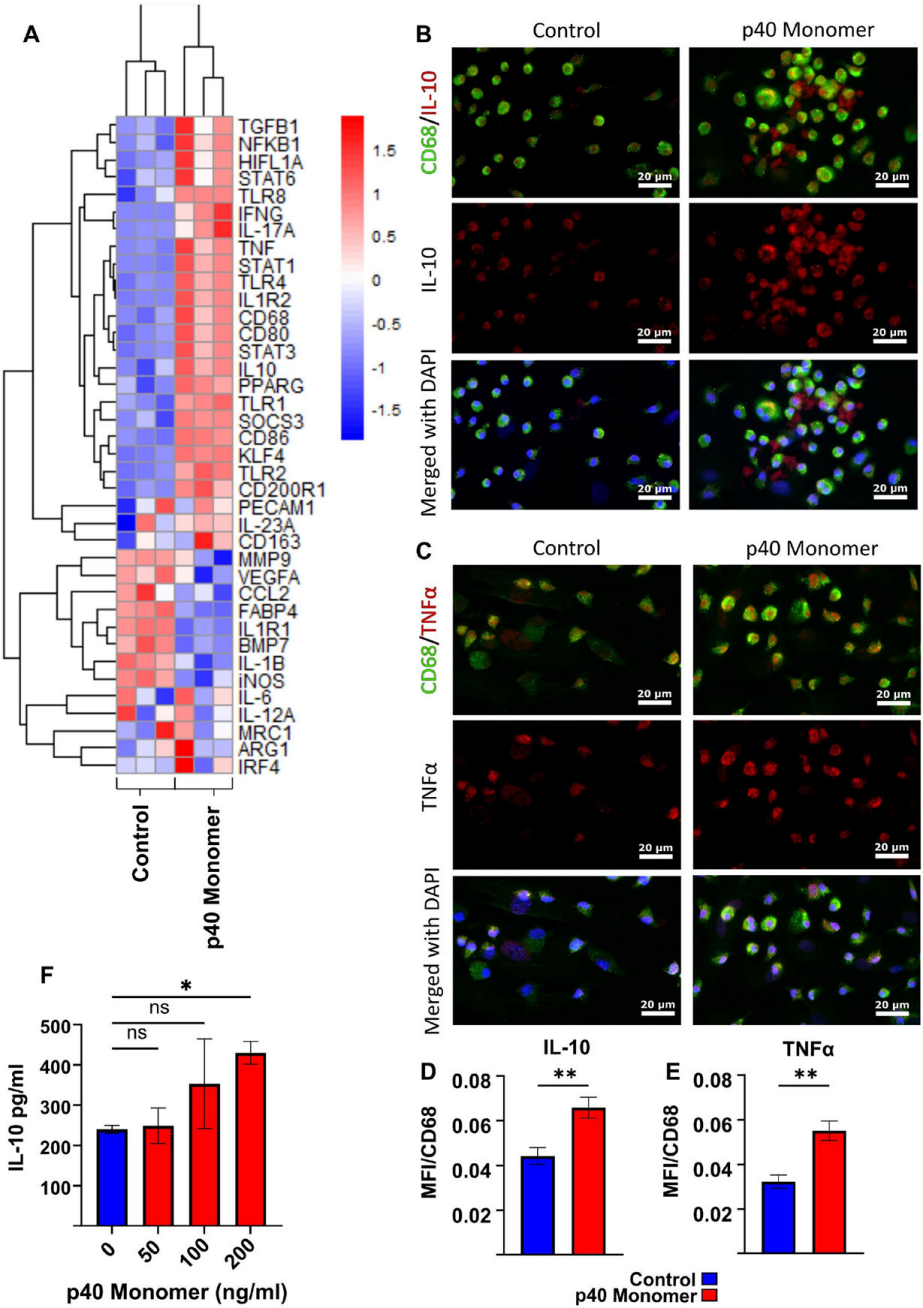
Effects of direct administration of recombinant p40 monomer on primary lung-associated macrophages. The heatmap shows transcriptomic changes via qPCR in lung-associated macrophages for a variety of relevant genes after 24 h treatment with 100 ng/mL p40 monomer on 70% confluent cells in serum-free medium (**A**). The immunofluorescent staining of lung-associated macrophages shows changes in levels of intracellular TNFα and IL-10 after 100 ng/mL p40 monomer treatment for 24 h (**B**,**C**). Quantitative MFI analysis was normalized to surface CD68 content to confirm that the signals measured were from macrophages (**D**,**E**). Primary cell culture supernatant was collected to measure IL-10 secretion levels following treatment with different concentrations of p40 monomer for 24 h via ELISA (**F**). Results are means ± SEMs of three different experiments. Significance was determined using one-way ANOVA analysis or two-tailed *t*-tests. * *p* < 0.05; ** *p* < 0.01; ns, not significant.

To determine whether the observed effects seen in the RAW 264.7 macrophage cell line could be replicated in primary cells, primary macrophages from adult mouse lungs were collected and treated with recombinant p40 monomer. To obtain a clearer picture of what effects p40 monomer has on lung primary cells, a panel of 39 genes associated with various macrophage subtypes were tested by qPCR analysis (Figure 2A). Results demonstrated significant changes in the transcriptomic profiles of lung macrophages treated with recombinant p40 monomer compared with those of the control set. Similar to the findings on RAW 264.7 cells, the results showed an increase in both pro- and anti-inflammatory markers. Some pro-inflammatory M1-like markers that were upregulated by p40 monomer include TNFα, CD86, and STAT1 (Figure 2A). However, anti-inflammatory M2-like markers such as IL-10, TGFβ, and STAT6 were also upregulated (Figure 2A). Consistently with what we found in RAW 264.7 cells, we found a decrease in iNOS (M1) and an increase in ARG1 (M2) after p40 monomer treatment (Figure 2A).

To confirm the findings at the protein level, immunofluorescent staining was performed to monitor intracellular levels of TNFα and IL-10 (Figure 2B-E). CD68 was used as a marker of macrophages. In line with our qPCR results, double-label immunofluorescence studies also showed upregulation of both IL-10 (Figure 2B) and TNFα (Figure 2C) in CD68-positive macrophages after p40 monomer treatment. These results were corroborated by quantification of mean fluorescence intensity (MFI) of IL-10 (Figure 2D) and TNFα (Figure 2E).

Finally, cell culture supernatants were collected to test for IL-10 production by sandwich ELISA (Figure 2F). Similar to the findings in RAW 264.7 cells, the results showed that p40 monomer dose-dependently increased the production of IL-10 in primary lung macrophages.

### 3.3. Recombinant p40 Monomer Attenuated LPS-Driven M1 Polarization in RAW 264.7 Cells and Primary Murine Lung Macrophages

**Figure 3.**
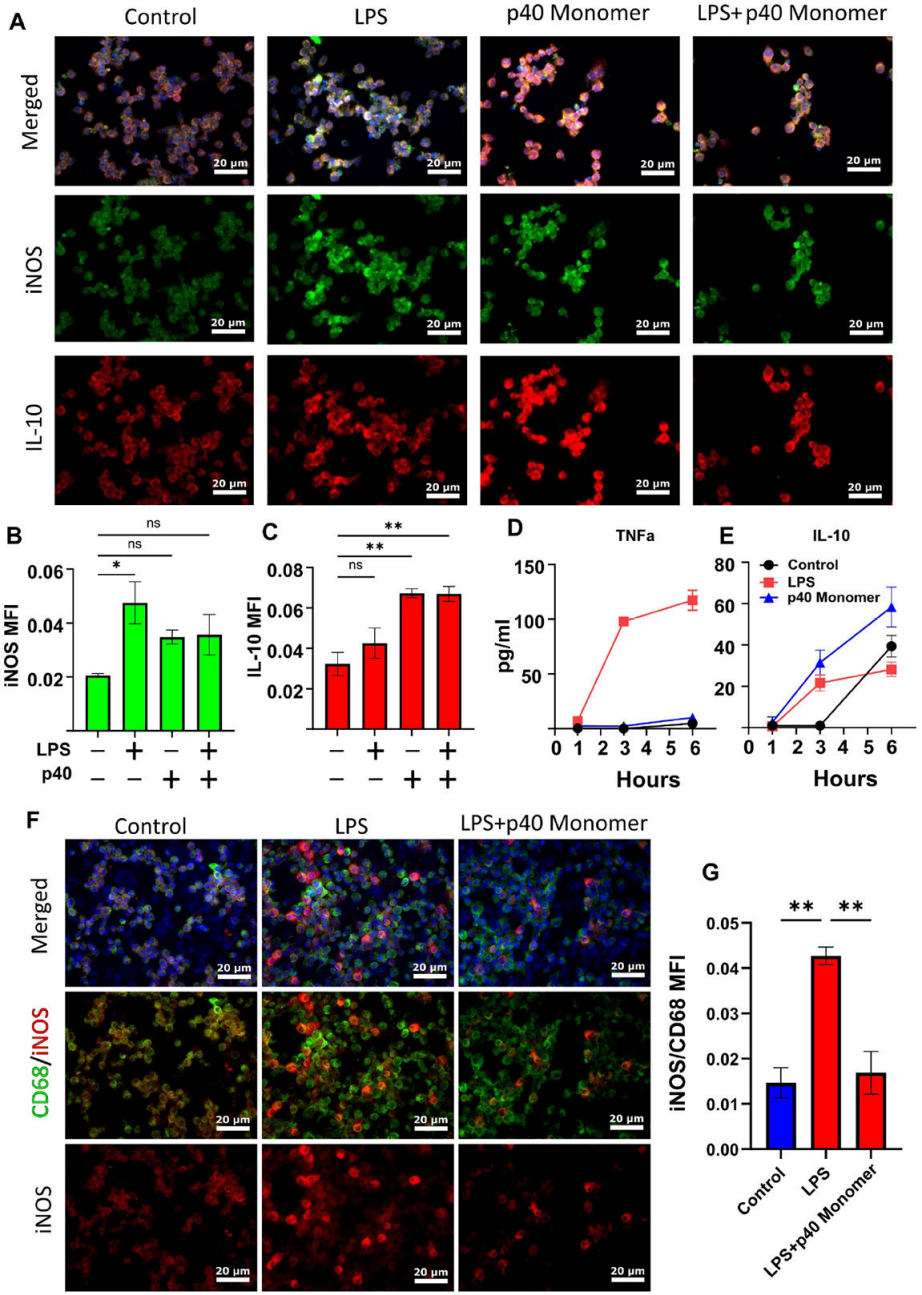
Effects of recombinant p40 monomer and LPS on RAW 264.7 cells and primary lung macrophages. RAW 264.7 cells were plated and pre-treated at 70% confluence with 100 ng/mL recombinant p40 monomer for 18 h and subsequently treated with 100 ng/mL LPS for 6 h in serum-free medium. Immunofluorescent staining shows levels of intracellular iNOS and IL-10 (**A**). Non-treated cells were considered controls (**A**). Quantitative MPI levels are presented as MPI per DAPI content (**B**,**C**). RAW 264.7 cells were treated at 70% confluence with either only LPS or p40 in serum-free medium for short-term 6 h treatment. Cell supernatants were collected for ELISA measurement of TNFα and IL-10 at 1, 3, and 6 h (**D**,**E**). Results are means ± SEMs of three different experiments. Primary lung macrophages were plated and treated for 6 h with LPS or pre-treated with p40 monomer for 18 h before 6 h LPS treatment. Immunofluorescent staining shows intracellular levels of iNOS and cell surface levels of CD68 (**F**). Quantitative analysis is presented as MFI normalized to CD68 content (**G**). All images were taken at 20× magnification. Significance was determined using one-way ANOVA analysis. * *p* < 0.05; ** *p* < 0.01; ns, not significant.

Our previous research showed that LPS treatment of primary peritoneal mouse macrophages and microglia resulted in a significant increase in the production of p40 monomer. With this in mind, we wanted to determine the role of p40 monomer with respect to M1 activation, specifically LPS/TLR4-induced M1 polarization [22]. RAW 264.7 cells were pre-treated with 100 ng/mL p40 monomer for 18 h and then stimulated with 100 ng/mL LPS for 6 h for a total of 24 h of treatment (Figure 3A). Immunofluorescence analysis predictably showed an increase in iNOS levels after LPS stimulation alone. Furthermore, in accordance with data shown earlier, the p40 monomer treatment alone exhibited a slight increase in iNOS levels (Figure 3B). However, after pre-treatment with p40 monomer, LPS challenge remained less effective in inducing iNOS compared with its non-pre-treated counterpart. Although expectedly [23], LPS increased the levels of IL-10 in primary lung macrophages, and p40 monomer was more potent than LPS in increasing the levels of IL-10 (Figure 3C). However, in the presence of both LPS and p40 monomer, the levels of IL-10 were found to be comparable with those in cells treated with p40 monomer only (Figure 3C).

To determine whether IL-10 secretion is a result of the direct administration of p40 monomer, and not a response to any inflammatory response, cells were plated for short-term treatment. Cells were treated for 1, 3, and 6 h before sample collection. LPS-treated cells were used for comparison. LPS-treated cells began to exhibit a large increase in TNFα secretion at 3 h, which further increased at 6 h (Figure 3D). On the other hand, p40 monomer-treated cells showed a slight but non-significant increase in TNFα within the 6 h window. When looking at IL-10 levels, both LPS- and p40 monomer-treated cells showed an increase after 3 h. However, our data showed that the levels of IL-10 obtained with p40 monomer treatment were higher than those obtained with LPS after treatment for different durations (Figure 3E). The results seem to indicate that the IL-10 release because of p40 monomer treatment was not due to any feedback response from TNFα or any effect of LPS treatment and that p40 monomer itself caused the increase.

To determine if these results were comparable in primary lung macrophages, double-label immunofluorescence was carried out to determine the level of M1 activation by LPS and whether p40 monomer could show any inhibitory effect. It was found that treatment with p40 monomer followed by LPS showed less iNOS production compared with its non-pre-treated counterpart. This may indicate that p40 monomer plays a role in blunting the inflammatory response to LPS in lungs (Figure 3F,G). Taken together, it may be the case that p40 monomer could play a significant role in either inhibiting M1 polarization or resolving the inflammatory effects of M1 macrophages.

### 3.4. mAb-Mediated Neutralization of p40 Monomer Resulted in Higher Levels of Pro-Inflammatory Cytokine Release and iNOS Expression in LPS-Treated RAW 264.7 Cells

**Figure 4.**
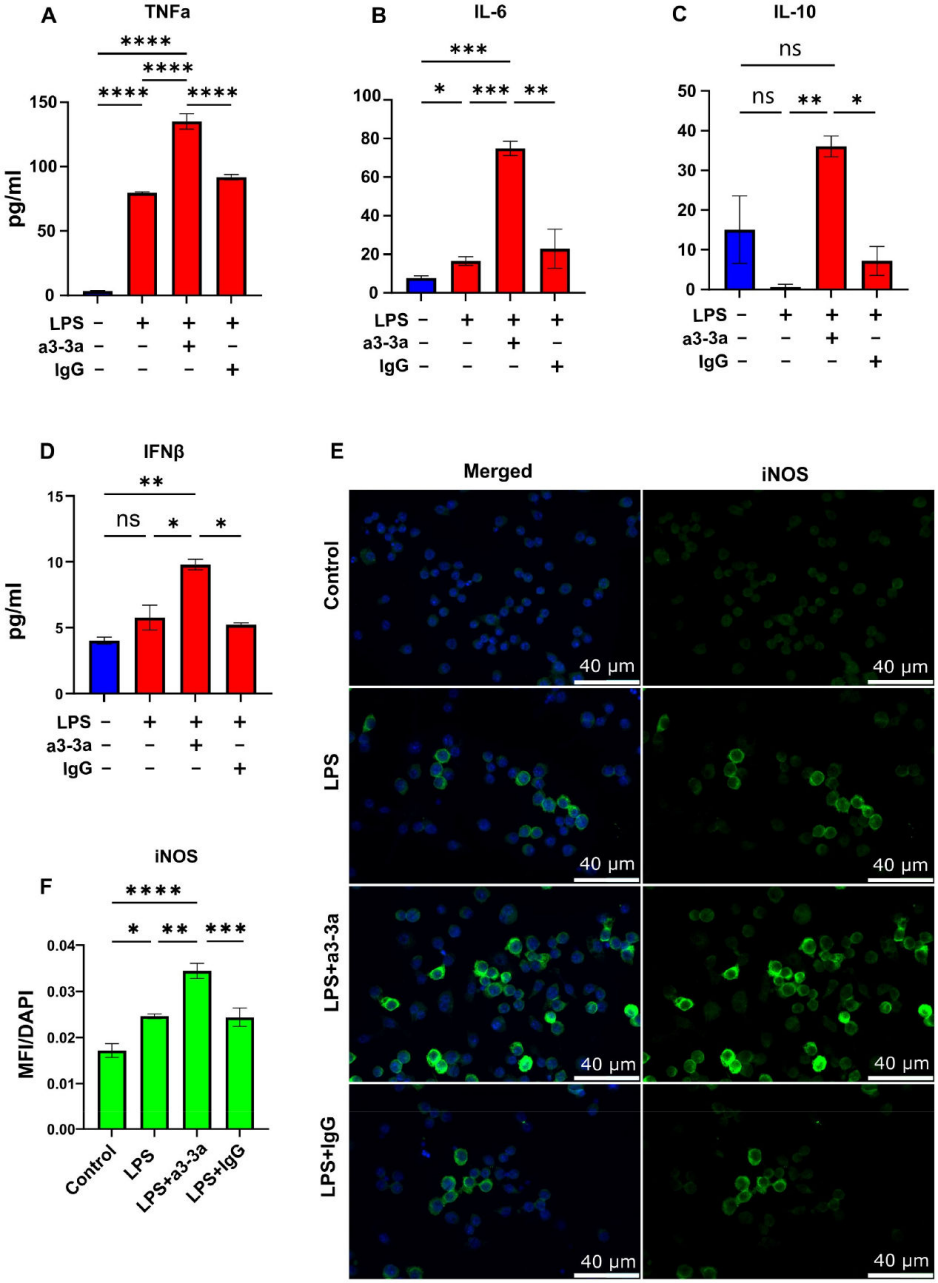
Effects of p40 monomer-neutralizing antibody a3-3a on LPS-treated RAW 264.7 cells. RAW 264.7 cells were treated at 70% confluency with 100 ng/mL LPS, LPS and 5 μg/mL of a3-3a, or LPS and 5 μg/mL of isotype hamster IgG in serum-free medium. Cells supernatants were collected 6 h after treatment for ELISA measurement of TNFα, IL-6, IL-10, and IFNβ (**A**–**D**). Immunofluorescent image analysis shows intracellular levels of iNOS with quantification presented as MFI per DAPI content (**E**,**F**). All images were taken at 40× magnification. Results are means ± SEMs of three different experiments. Significance was determined using one-way ANOVA analysis. * *p* < 0.05; ** *p* < 0.01; *** *p* < 0.001; **** *p* < 0.0001; ns, not significant.

To further explore whether p40 monomer plays a role in macrophage regulation, our novel monoclonal antibodies (a3-3a) [18] were used to neutralize the effects of p40 monomer in LPS-treated cultures. Cells were treated concurrently with 100 ng/mL LPS and 5 μg/mL a3-3a antibody for 6 h. As control, cells were also treated with hamster IgG. ELISA results showed a significant increase in TNFα release (Figure 4A) and IL-6 (Figure 4B), demonstrating that the neutralization of p40 monomer led to greater secretion of these cytokines compared with that of LPS alone. However, when measuring the levels of IL-10 (Figure 4C), it was found that treatment with both LPS and a3-3a also induced a significant increase. This is possibly due to the macrophage response to LPS, in which there is upregulation of type I interferons capable of inducing IL-10 production [24,25]. Indeed, while analyzing the level of type I interferon IFNβ by ELISA, we found a significant increase in levels after treatment with both LPS and a3-3a (Figure 4D). Furthermore, the immunofluorescence results (Figure 4E,F) show a significant increase in iNOS-positive macrophages, indicating that in the absence of any potential autocrine regulatory effects of p40 monomer, LPS-driven M1 polarization of macrophages exhibits a stronger pro-inflammatory response, followed by a faster resolution mediated by IFNβ.

### 3.5. Intranasal Administration of Recombinant p40 Monomer Resulted in Increased Levels of CD68^+^TNFα^+^IL-10^+^ Macrophages In Vivo in the Lungs of Mice

**Figure 5.**
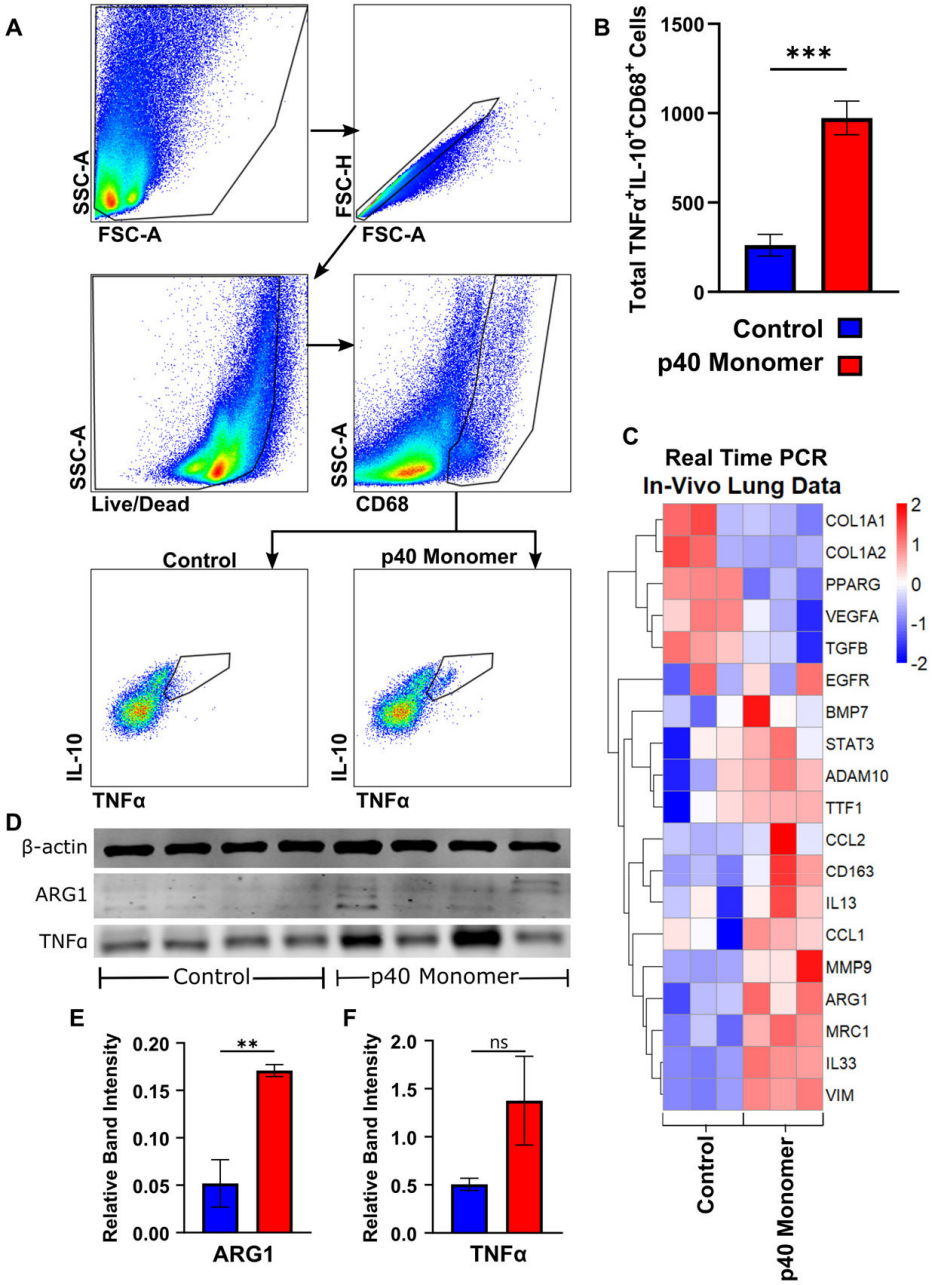
Effects of intranasal administration of recombinant p40 monomer on C57BL/6 wild-type mice. C57BL/6 mice (n = 4) were treated intranasally with 50 ng p40 monomer daily for 4 days. Sterile filtered DPBS was administered to control mice. On day 4, lung tissue was harvested. The flow cytometric gating strategy isolates live single cells and CD68^+^ cells from lung single cells. TNFα and IL-10 double-positive cells were measured (**A**). The total number of CD68^+^TNFα^+^IL-10^+^ cells was compared between control and rp40 monomer-treated mouse lungs (**B**). The heatmap shows differences in the transcriptomic levels of the chosen markers (**C**). The immunoblot analysis of protein extracted from lung tissue demonstrates levels of TTF-1, ARG1, and TNFα, with β-actin as loading control (**D**). Quantification measure band intensity of target normalized to intensity of β-actin (**E**–**F**). Results are means ± SEMs of four mice per group. Significance was determined using two-tailed *t*-tests. ** *p* < 0.01; *** *p* < 0.001; ns, not significant.

To determine whether the effects seen in cell cultures could be translated in vivo, C57/BL6 mice were treated with p40 monomer intranasally, and single-cell suspension from the lungs were stained for CD68 by FACS to select macrophages along with TNFα and IL-10. Our initial trial experiments demonstrated that recombinant p40 monomer at a dose of 50 ng/mouse was sufficient to induce a measurable change in the lung-associated macrophage phenotype. The results showed a significant increase in TNFα^+^IL-10^+^ macrophages induced by p40 monomer as compared with the PBS control (Figure 5A,B).

Real-time qPCR was run for specific gene targets from lung tissue mRNA (Figure 5C). Results from these runs showed a decreasing trend in transcripts associated with a tumorigenic phenotype, such as TGFβ and VEGFa, while there were no noticeable changes in others, such as EGFR. In addition, we noticed an increase in ADAM10, which has been shown to be associated with NSCLC and a potential biomarker for lung cancer detection [26,27]. We also observed increases in certain M2-associated genes (ARG1, CD163, STAT3, and MRC1), markers associated with tissue remodeling (VIM and MMP9), and Th2-associated markers (IL-13, IL-33, CCL1, and CCL2) after p40 monomer treatment.

The Western blot analysis of whole lung tissue also showed a significant increase in the levels of ARG1 (Figure 5E) and TNFα (Figure 5F), although not at the level to reach significance in the case of the latter one. The increase in ARG1 levels in the lung, combined with the transcriptomic upregulation of markers associated with Th2 response and lung remodeling, may indicate that p40 monomer may play a role not only in resolving inflammation but also in the remodeling phase associated with post injury/inflammation.

## 4. Discussion

Macrophages play a multitude of roles, such as mediating inflammatory responses, defense against pathogens, or homeostatic functions of tissue maintenance [28]. Macrophages and signals associated with macrophages can be used as diagnostic markers and targets for treatment of certain diseases via modulating their surrounding microenvironment [29]. Signals such as LPS and/or IFNγ stimulate an M1 phenotype, while signals such as IL-4 and IL-13 stimulate an M2 phenotype. However, rather than existing in a distinct M1 or M2 state, studies report that macrophages exist on more of a spectrum, with the identification of subtypes such as M1, M2a, M2b, M2c, or M2d, which are capable of producing a combination of signals classically considered pro- or anti-inflammatory [4,12,30].

While M1 macrophages are pro-inflammatory and express high amounts of iNOS, M2 macrophages are anti-inflammatory and characterized by ARG1. Therefore, increases in ARG1 and decreases in iNOS induced by p40 monomer may seemingly indicate polarization of macrophages towards the M2 phenotype. However, IL-10 is an important anti-inflammatory regulatory effector cytokine produced by M2 macrophages, and our data suggest that p40 monomer alone is sufficient to stimulate not only IL-10 release but also TNFα and IL-6, both classically considered pro-inflammatory. With this paradoxical pattern of effector cytokine release, it is difficult to categorize these cells as either wholly pro-inflammatory (M1) or anti-inflammatory (M2). While seemingly contradictory in nature, there have been reports of this pattern of cytokine release. For example, a subset of M2 macrophages known as M2b macrophages have been shown to secrete a combination of pro- and anti-inflammatory cytokines. Activated by combinations of LPS, immune complexes, or IL-1β, one of the most characteristic markers of this subtype is CCL1 [31,32]. In fact, we see from our RAW 264.7 cell culture and in vivo lung qPCR panel that CCL1 is upregulated in the presence of p40 monomer. Therefore, p40 monomer may be responsible for the generation of M2b macrophages. Moreover, recently, we have seen that p40 monomer suppresses the disease process of EAE in mice [11]. Since IL-10 is a key anti-inflammatory cytokine for the suppression of autoimmune diseases like MS [33,34], it is likely that IL-10 plays a role in the p40 monomer-mediated protection of EAE.

We also observe a similar pattern of expression of both pro- and anti-inflammatory profiles in primary lung macrophages, especially when considering TNFα and IL-10. While lungs contain a heterogeneous population of macrophages [35], our data seem to imply that p40 monomer is capable of modulating any macrophage of monocytic lineage. This, combined with our RAW 264.7 cell data and our previously published work on microglia [36], implies some degree of ubiquity in p40 monomer’s effects on different populations of macrophages in the body. This is significant when considering that studies have shown correlative predictive effects for various conditions, such as coronary artery disease and infections associated with burn severities using TNFα/IL-10 ratios [37-39]. The data presented could give insight into how macrophages and their surrounding tissue regulate this ratio and give stronger evidence of p40 monomer as a possible prognostic biomarker for various diseases [40-42].

Here, we avoided using knockout models (e.g., p40 null mice that are expected to be deficient in IL-12, 1L-23, p40 homodimer, and p40 monomer) and investigated the direct effects of p40 monomer in the presence of LPS. Indeed, in the presence of p40 monomer, LPS did not elicit as strong of an M1 response as it did alone. This may be due to a regulatory function of p40 monomer in macrophages. One way by which p40 monomer may play a regulatory role is the early release of IL-10 to mitigate the effects of LPS. It has been demonstrated in the past that classically activated macrophages have intrinsic ways of self-limiting its inflammatory characteristics [23,43,44]. Considering our previous report that LPS treatment alone was enough to elicit an p40 monomer release in peritoneal macrophages and microglia [18], this may demonstrate that p40 monomer acts as another intermediate player between LPS and IL-10, and that in the presence of p40 monomer, macrophages are already primed to produce IL-10, thus blunting the inflammatory response of LPS stimulation. This was further supported by a stronger LPS-induced M1 pro-inflammatory signal in the presence of a neutralizing mAb against p40 monomer. Therefore, p40 monomer plays an important role in the immunosuppression and/or self-regulation of macrophages.

Another important finding is the upregulation of CD68^+^TNFα^+^IL-10^+^ macrophages in the lungs after p40 monomer treatment. At this stage, it is unclear what effect these cells may have on the overall function within the lungs, but clues may be found in our qPCR panel data. We found a phenotypic profile consistent with M2-like response, which was supported by our immunoblot results confirming the upregulation of ARG1. M2 and Th2 markers (e.g., IL-13 and IL-33), along with markers, such as vimentin, that were found to be upregulated, seemed to be indicative of a shift towards an environment conducive to lung remodeling. It is known that M2 and Th2 responses are often associated with tissue remodeling and wound healing, especially in disorders such as allergy, asthma, and inflammation [45-47]. While we found that certain matrix protein (e.g., COL1A1 and COL1A2) transcript levels decreased after p40 monomer treatment, we did see a clear increase in vimentin. Vimentin, often a marker of epithelial-to-mesenchymal transition (EMT), has been found to be associated with the onset of fibrosis [48], and studies have shown that macrophages facilitate repair via vimentin [49].

## 5. Conclusions

In summary, we have demonstrated that p40 monomer is a possible player in the immune regulation of macrophages and lung tissue remodeling by demonstrating that (1) p40 monomer directly induces production of both TNFα and IL-10 in macrophages, (2) p40 monomer potentially regulates M1 polarization, and (3) direct administration of p40 monomer through the lungs may facilitate M2/Th2-driven lung remodeling.

## Data Availability

All data are present in this manuscript.

## References

[1] Mosser DM; Edwards JP Exploring the full spectrum of macrophage activation. Nat. Rev. Immunol 2008, 8, 958–969. 19029990 10.1038/nri2448 PMC2724991

[2] Murray PJ; Wynn TA Protective and pathogenic functions of macrophage subsets. Nat. Rev. Immunol 2011, 11, 723–737. 21997792 10.1038/nri3073 PMC3422549

[3] Shapouri-Moghaddam A; Mohammadian S; Vazini H; Taghadosi M; Esmaeili SA; Mardani F; Seifi B; Mohammadi A; Afshari JT; Sahebkar A Macrophage plasticity, polarization, and function in health and disease. J. Cell. Physiol 2018, 233, 6425–6440. 10.1002/jcp.26429 PMC13482560 29319160

[4] Murray PJ Macrophage Polarization. Annu. Rev. Physiol 2017, 79, 541–566. 27813830 10.1146/annurev-physiol-022516-034339

[5] Ochando J; Mulder WJM; Madsen JC; Netea MG; Duivenvoorden R Trained immunity—Basic concepts and contributions to immunopathology. Nat. Rev. Nephrol 2023, 19, 23–37. 36253509 10.1038/s41581-022-00633-5 PMC9575643

[6] Orecchioni M; Ghosheh Y; Pramod AB; Ley K Macrophage Polarization: Different Gene Signatures in M1(LPS+) vs. Classically and M2(LPS−) vs. Alternatively Activated Macrophages. Front. Immunol 2019, 10, 1084. 31178859 10.3389/fimmu.2019.01084 PMC6543837

[7] Yunna C; Mengru H; Lei W; Weidong C Macrophage M1/M2 polarization. Eur. J. Pharmacol 2020, 877, 173090. 32234529 10.1016/j.ejphar.2020.173090

[8] Tang PM; Nikolic-Paterson DJ; Lan HY Macrophages: Versatile players in renal inflammation and fibrosis. Nat. Rev. Nephrol 2019, 15, 144–158. 30692665 10.1038/s41581-019-0110-2

[9] Cheng P; Li S; Chen H Macrophages in Lung Injury, Repair, and Fibrosis. Cells 2021, 10, 436. 33670759 10.3390/cells10020436 PMC7923175

[10] Boutilier AJ; Elsawa SF Macrophage Polarization States in the Tumor Microenvironment. Int. J. Mol. Sci 2021, 22, 6995. 34209703 10.3390/ijms22136995 PMC8268869

[11] Mondal S; Kundu M; Jana M; Roy A; Rangasamy SB; Modi KK; Wallace J; Albalawi YA; Balabanov R; Pahan K IL-12 p40 monomer is different from other IL-12 family members to selectively inhibit IL-12Rbeta1 internalization and suppress EAE. Proc. Natl. Acad. Sci. USA 2020, 117, 21557–21567. 32817415 10.1073/pnas.2000653117 PMC7474649

[12] Wang L-X; Zhang S-X; Wu H-J; Rong X-L; Guo J M2b macrophage polarization and its roles in diseases. J. Leukoc. Biol 2019, 106, 345–358. 30576000 10.1002/JLB.3RU1018-378RR PMC7379745

[13] Benveniste EN Inflammatory cytokines within the central nervous system: Sources, function, and mechanism of action. Am. J. Physiol 1992, 263, C1–C16. 1636671 10.1152/ajpcell.1992.263.1.C1

[14] Gately MK; Renzetti LM; Magram J; Stern AS; Adorini L; Gubler U; Presky DH The interleukin-12/interleukin-12-receptor system: Role in normal and pathologic immune responses. Annu. Rev. Immunol 1998, 16, 495–521. 9597139 10.1146/annurev.immunol.16.1.495

[15] Gaffen SL; Jain R; Garg AV; Cua DJ The IL-23-IL-17 immune axis: From mechanisms to therapeutic testing. Nat. Rev. Immunol 2014, 14, 585–600. 25145755 10.1038/nri3707 PMC4281037

[16] Cua DJ; Sherlock J; Chen Y; Murphy CA; Joyce B; Seymour B; Lucian L; To W; Kwan S; Churakova T; Interleukin-23 rather than interleukin-12 is the critical cytokine for autoimmune inflammation of the brain. Nature 2003, 421, 744–748. 12610626 10.1038/nature01355

[17] Gillessen S; Carvajal D; Ling P; Podlaski FJ; Stremlo DL; Familletti PC; Gubler U; Presky DH; Stern AS; Gately MK Mouse interleukin-12 (IL-12) p40 homodimer: A potent IL-12 antagonist. Eur. J. Immunol 1995, 25, 200–206. 7843232 10.1002/eji.1830250133

[18] Dasgupta S; Bandopadhyay M; Pahan K Generation of functional blocking monoclonal antibodies against mouse interleukin-12 p40 homodimer and monomer. Hybridoma 2008, 27, 141–151. 18582206 10.1089/hyb.2007.0560 PMC2673812

[19] Mondal S; Roy A; Pahan K Functional blocking monoclonal antibodies against IL-12p40 homodimer inhibit adoptive transfer of experimental allergic encephalomyelitis. J. Immunol 2009, 182, 5013–5023. 19342681 10.4049/jimmunol.0801734 PMC2721330

[20] Kundu M; Raha S; Roy A; Pahan K Regression of Triple-Negative Breast Cancer in a Patient-Derived Xenograft Mouse Model by Monoclonal Antibodies against IL-12 p40 Monomer. Cells 2022, 11, 259. 35053375 10.3390/cells11020259 PMC8773899

[21] Kundu M; Roy A; Pahan K Selective neutralization of IL-12 p40 monomer induces death in prostate cancer cells via IL-12-IFN-gamma. Proc. Natl. Acad. Sci. USA 2017, 114, 11482–11487. 29073075 10.1073/pnas.1705536114 PMC5664500

[22] Park BS; Lee J-O Recognition of lipopolysaccharide pattern by TLR4 complexes. Exp. Mol. Med 2013, 45, e66. 24310172 10.1038/emm.2013.97 PMC3880462

[23] Pengal RA; Ganesan LP; Wei G; Fang H; Ostrowski MC; Tridandapani S Lipopolysaccharide-induced production of interleukin-10 is promoted by the serine/threonine kinase Akt. Mol. Immunol 2006, 43, 1557–1564. 16263172 10.1016/j.molimm.2005.09.022

[24] Kumaran Satyanarayanan S; El Kebir D; Soboh S; Butenko S; Sekheri M; Saadi J; Peled N; Assi S; Othman A; Schif-Zuck S; IFN-beta is a macrophage-derived effector cytokine facilitating the resolution of bacterial inflammation. Nat. Commun 2019, 10, 3471. 31375662 10.1038/s41467-019-10903-9 PMC6677895

[25] Karimi Y; Giles EC; Vahedi F; Chew MV; Nham T; Loukov D; Lee AJ; Bowdish DM; Ashkar AA IFN-beta signalling regulates RAW 264.7 macrophage activation, cytokine production, and killing activity. Innate Immun. 2020, 26, 172–182. 31615311 10.1177/1753425919878839 PMC7144030

[26] Guo J; He L; Yuan P; Wang P; Lu Y; Tong F; Wang Y; Yin Y; Tian J; Sun J ADAM10 overexpression in human non-small cell lung cancer correlates with cell migration and invasion through the activation of the Notch1 signaling pathway. Oncol. Rep 2012, 28, 1709–1718. 22940701 10.3892/or.2012.2003

[27] Yoneyama T; Gorry M; Sobo-Vujanovic A; Lin Y; Vujanovic L; Gaither-Davis A; Moss ML; Miller MA; Griffith LG; Lauffenburger DA; ADAM10 Sheddase Activity is a Potential Lung-Cancer Biomarker. J. Cancer 2018, 9, 2559–2570. 30026855 10.7150/jca.24601 PMC6036891

[28] Hirayama D; Iida T; Nakase H The Phagocytic Function of Macrophage-Enforcing Innate Immunity and Tissue Homeostasis. Int. J. Mol. Sci 2017, 19, 92. 29286292 10.3390/ijms19010092 PMC5796042

[29] Zhang C; Yang M; Ericsson AC Function of Macrophages in Disease: Current Understanding on Molecular Mechanisms. Front. Immunol 2021, 12, 620510. 33763066 10.3389/fimmu.2021.620510 PMC7982479

[30] Li P; Ma C; Li J; You S; Dang L; Wu J; Hao Z; Li J; Zhi Y; Chen L; Proteomic characterization of four subtypes of M2 macrophages derived from human THP-1 cells. J. Zhejiang Univ. Sci. B 2022, 23, 407–422. 35557041 10.1631/jzus.B2100930 PMC9110321

[31] Asai A; Nakamura K; Kobayashi M; Herndon DN; Suzuki F CCL1 released from M2b macrophages is essentially required for the maintenance of their properties. J. Leukoc. Biol 2012, 92, 859–867. 22730547 10.1189/jlb.0212107

[32] Asai A; Tsuchimoto Y; Ohama H; Fukunishi S; Tsuda Y; Kobayashi M; Higuchi K; Suzuki F Host antitumor resistance improved by the macrophage polarization in a chimera model of patients with HCC. Oncoimmunology 2017, 6, e1299301. 28507807 10.1080/2162402X.2017.1299301 PMC5414886

[33] Guo B. IL-10 Modulates Th17 Pathogenicity during Autoimmune Diseases. J. Clin. Cell. Immunol 2016, 7, 400. 27308096 10.4172/2155-9899.1000400 PMC4905582

[34] Spach KM; Nashold FE; Dittel BN; Hayes CE IL-10 signaling is essential for 1,25-dihydroxyvitamin D3-mediated inhibition of experimental autoimmune encephalomyelitis. J. Immunol 2006, 177, 6030–6037. 17056528 10.4049/jimmunol.177.9.6030

[35] Cai Y; Sugimoto C; Arainga M; Alvarez X; Didier ES; Kuroda MJ In vivo characterization of alveolar and interstitial lung macrophages in rhesus macaques: Implications for understanding lung disease in humans. J. Immunol 2014, 192, 2821–2829. 24534529 10.4049/jimmunol.1302269 PMC3959879

[36] Jana M; Dasgupta S; Saha RN; Liu X; Pahan K Induction of tumor necrosis factor-alpha (TNF-alpha) by interleukin-12 p40 monomer and homodimer in microglia and macrophages. J. Neurochem 2003, 86, 519–528. 12871593 10.1046/j.1471-4159.2003.01864.x PMC1955470

[37] Goswami B; Rajappa M; Mallika V; Shukla DK; Kumar S TNF-alpha/IL-10 ratio and C-reactive protein as markers of the inflammatory response in CAD-prone North Indian patients with acute myocardial infarction. Clin. Chim. Acta 2009, 408, 14–18. 19576194 10.1016/j.cca.2009.06.029

[38] Kumari R; Kumar S; Ahmad MK; Singh R; Pradhan A; Chandra S; Kumar S TNF-alpha/IL-10 ratio: An independent predictor for coronary artery disease in North Indian population. Diabetes Metab. Syndr 2018, 12, 221–225. 28988596 10.1016/j.dsx.2017.09.006

[39] Tsurumi A; Que YA; Ryan CM; Tompkins RG; Rahme LG TNF-alpha/IL-10 Ratio Correlates with Burn Severity and May Serve as a Risk Predictor of Increased Susceptibility to Infections. Front. Public Health 2016, 4, 216. 27761434 10.3389/fpubh.2016.00216 PMC5050217

[40] Bai M; Zhang J; Su X; Yao X; Li H; Cheng J; Mao J; Li X; Chen J; Lin W Serum IL-12p40: A novel biomarker for early prediction of minimal change disease relapse following glucocorticoids therapy. Front. Med 2022, 9, 922193. 10.3389/fmed.2022.922193 PMC9729255 36507530

[41] Shawky H; El-Shenawy R; Helmy NM Circulating macrophage inflammatory protein-1β/IL-12p40 ratio predicts sofosbuvir-based treatment outcome in HCV- genotype 4 patients. Hum. Antibodies 2021, 29, 263–274. 34511496 10.3233/HAB-211504

[42] Stanilov N; Miteva L; Jovchev J; Cirovski G; Stanilova S The prognostic value of preoperative serum levels of IL-12p40 and IL-23 for survival of patients with colorectal cancer. APMIS 2014, 122, 1223–1229. 24909386 10.1111/apm.12288

[43] Alexander AF; Kelsey I; Forbes H; Miller-Jensen K Single-cell secretion analysis reveals a dual role for IL-10 in restraining and resolving the TLR4-induced inflammatory response. Cell Rep. 2021, 36, 109728. 34551303 10.1016/j.celrep.2021.109728 PMC8995750

[44] Iyer SS; Ghaffari AA; Cheng G Lipopolysaccharide-mediated IL-10 transcriptional regulation requires sequential induction of type I IFNs and IL-27 in macrophages. J. Immunol 2010, 185, 6599–6607. 21041726 10.4049/jimmunol.1002041 PMC4103176

[45] Hough KP; Curtiss ML; Blain TJ; Liu RM; Trevor J; Deshane JS; Thannickal VJ Airway Remodeling in Asthma. Front. Med 2020, 7, 191. 10.3389/fmed.2020.00191 PMC7253669 32509793

[46] Iwasaki N; Terawaki S; Shimizu K; Oikawa D; Sakamoto H; Sunami K; Tokunaga F Th2 cells and macrophages cooperatively induce allergic inflammation through histamine signaling. PLoS ONE 2021, 16, e0248158. 33662037 10.1371/journal.pone.0248158 PMC7932145

[47] Koh TJ; DiPietro LA Inflammation and wound healing: The role of the macrophage. Expert Rev. Mol. Med 2011, 13, e23. 21740602 10.1017/S1462399411001943 PMC3596046

[48] Surolia R; Li FJ; Wang Z; Li H; Dsouza K; Thomas V; Mirov S; Pérez-Sala D; Athar M; Thannickal VJ; Vimentin intermediate filament assembly regulates fibroblast invasion in fibrogenic lung injury. JCI Insight 2019, 4, e123253. 30944258 10.1172/jci.insight.123253 PMC6483650

[49] Mor-Vaknin N; Punturieri A; Sitwala K; Markovitz DM Vimentin is secreted by activated macrophages. Nat. Cell Biol 2003, 5, 59–63. 12483219 10.1038/ncb898

